# Frequency and risk factors of *H. pylori* infection among dental students: an observational cross-sectional study

**DOI:** 10.1038/s41598-023-41246-7

**Published:** 2023-08-31

**Authors:** Christine Raouf George Mikhail, Abeer Abd El Maksoud Mohamed, Olfat Gamil Shaker, Eman EL Desouky, Rania Hassan Shalaby

**Affiliations:** 1 Oral Medicine and Diagnosis, Department of Oral Medicine, Diagnosis and Periodontology, Faculty of Dentistry, Fayoum University, Fayoum, Egypt; 2 Oral Biology, Department of Oral Biology, Faculty of Dentistry, Fayoum University, Fayoum, Egypt; 3 Medical Biochemistry and Molecular Biology, Faculty of Medicine, Cairo University, Cairo, Egypt; 4 Epidemiology and Biostatistics, NCI, Cairo University, Cairo, Egypt

**Keywords:** Health care, Health occupations, Medical research, Risk factors

## Abstract

Despite *Helicobacter pylori* infection remains asymptomatic in most people, it is associated with an increased risk of gastric cancer. Considering Egypt had the highest prevalence of *H. pylori* in healthy asymptomatic population in adults and pediatric age in past studies and currently salivary ELISA could be used for diagnosis of Oral *H. pylori* infection. Moreover, some researchers speculated that dentists and dental students might be at a higher risk for oral *H. pylori* infection because they are the most frequently exposed ones to saliva and dental plaqu*e.* This study aimed to determine risk factors associated with frequency of *H. pylori* among a sample of dental students for better management of the disease. 83 participants, with age (21–25 years), attending Faculty of Dentistry, Fayoum University were recruited. A structured questionnaire was used to collect information on sociodemographic parameters and risk factors for *H. pylori*. Direct inquiry about dyspeptic symptoms were done. Saliva samples were collected and tested for H. pylori antibodies. Overall seroprevalence was 22.9%. Participants in internship were more prone to be positive (*p* = 0.005). 32.6% of urban residents versus 10.8% of rural were *H. pylori* positive (*p* = 0.019). 75.0% of previous history of *H. pylori* infection versus 14.1% of those with no history were *H. pylori* positive *p* < 0.001. 70% of positive *H. pylori* participants reported positive clinical symptoms that were statistically significant. This study suggests that middle income, previous history of *H. pylori* and clinical symptoms of dyspepsia are risk factors of oral *H. pylori* with a decline in its prevalence in Egypt.

## Introduction

The microaerophilic, rod shaped, gram negative, Campylobacterales member Helicobacter pylori *(H. pylori)* is causative agent and key factor in the development of gastritis, gastroduodenal ulcers, and gastric cancer^1^. *H. pylori* is one of the primary causes of infection-attributable cancer cases worldwide in accordance to a sub-analysis of the 2018 Global Burden of Disease survey^2^. Martel et al.^3^ emphasized that most individuals will likely get *H. pylori* at least once throughout their lives.

Despite the exact route of transmission of *H. pylori* is not exactly known, it is considered to be contagious^4^. In addition, researchers have been concerned with the presence of *H. pylori* in the oral cavity since it is the first component of the gastrointestinal system. Some researchers even speculated that oral-oral transmission route is the most likely transmission pathway because DNA of *H. pylo*ri had been detected in several body fluids, including vomit, dental plaque, saliva and gastric secretions^1^. Accordingly, the oral cavity may act as a reservoir for *H. pylori* bacteria. For this reason, its detection in oral cavity samples has been recommended as a diagnostic test^5^.

Considering dentists are the most frequently exposed ones to the infected oral contents such as saliva and dental plaque, therefore studies were done to compare the detection frequency of oral H. pylori among dentists. Liu et al.^6^ observed a higher *H. pylori* frequency among the dentists than the nondentists. Also, Lin et al.^7^ that the prevalence of *H. pylori* infection in dentists, dental nurses, fifth year dental students and first year dental students were 23%, 18%, 18%and 16%, respectively. They concluded that dentists are at a higher risk for *H. pylori* infection, and intensive attention should be paid to this issue.

Evaluating and correlating the presence of *H. pylori* antibodies from the saliva of three groups (30 infants, 30 adolescents (dental students), and 30 elderly individuals) with varied living conditions. Infants showed 23 positive results, 3 negative results and 4 equivalent. Age group between 20 and 25 showed 25 positive results, 2 negative results and 3 equivalent and age group 50 and above showed 25 positive results, 1 negative results and 4 equivalent^8^. This observation speculated that dental students age group between 20 and 25 could be at a higher risk for oral *H. pylori* infection.

Once the gastric mucosa became colonized by *H. pylori*, its persistence could be for a lifetime, and fortunately, the immune system was able to tolerate its existence. Moreover, some favour its persistence in the stomach, but others don’t. Those with high and extremely low prevalence of *H. pylori* could provide useful insights on the clinical outcomes associating this infection. Adverse effects including peptic ulcer and gastric cancer depend on a delicate balance between an innocent inflammation and a severe inflammation^9^.

Noninvasive serologic and saliva diagnostic assays for antibodies to *H. pylori* are available in addition to histologic detection of the pathogen. Studies have shown that the levels of circulating immunoglobulins are almost equivalent to those of salivary immunoglobulins^10^. Moreover, salivary ELISA has diagnostic values comparable to serum ELISA, suggesting it might be utilized as a substitute for the latter in the diagnosis of *H. pylori* infection^11^.

The rate of *H. pylori* infection varies widely depending on demographic factors such as age, location, race, socioeconomic status, blood group O, high body mass index, poor hygiene, smoking, crowded living situations, the use of nonsteroidal anti-inflammatory drug (NSAID), and family history of gastric illness^4^. Almost over two-thirds of the world’s population is infected with *H. pylori* with rates as high as 80% seen in developing nations. In contrast, in industrialized nations, prevalence is declining, often to about 40%. Despite of that the majority of *H. pylori* infection are asymptomatic, the presence of these bacteria is linked to an elevated risk of many gastric illness, mostly notably cancer^12^.

According to Egyptological research on *H. pylori*, Egypt has the world’s highest prevalence of *H. pylori* among asymptomatic individuals of all ages^13–15^. Furthermore, the prevalence of *H. pylori* in Egypt was thought to rise in tandem with the severity of socioeconomic disadvantage, low bodymass index (BMI), rural residence and lack of formal education^16^. A population-based cross-sectional study performed among asymptomatic school children reported that the overall *H. pylori* prevalence was 72.38%. Its main risk factors are residing in an overcrowded home and socially deprived area^14^. Mothers of *H. pylori*-infected children showed higher seroprevalence (39.5%) than their siblings and fathers (37.7% and 22.8%, respectively). In a rural area, relatives with low socioeconomic level generally showed the highest seroprevalence (82.5% and 78.1%, respectively)^17^.

Another cross-sectional study found that *H. pylori* seroprevalence was significantly age-dependent: 60.6% of patients aged more than 5 years and 25.9% of patients aged less than 5 years. One of the main risk factors associated with seroprevalence was crowding in beds. The seroprevalence among children was 59.7% in case of more than 3 persons sharing a bed and 26.9% in case of fewer than 3 persons sharing a bed. A cross-sectional study carried out in Tanta City in the Nile Delta reported prevalence of about 70%, indicating that the burden of *H. pylori* infection is high in rural areas than in urban areas^18^.

Epidemiological studies showed a strong association between *H. pylori* and many gastroduodenal and extra-gastroduodenal diseases, namely dyspepsia, peptic ulcer, gastric cancer, and the gastric marginal zone B-cell lymphoma of mucosa-associated lymphoid tissue^19^. Moreover, cumulative evidence showed that patients on long-term antithrombotic therapy (including low-dose aspirin and long-term anticoagulation) or initiating long-term nonsteroidal antiinflammatory drug therapy should be tested for *H. pylori* infection and treated if tested positive to reduce the risk of ulcer bleeding^20^. Evidence also suggested that eradication of *H. pylori* could improve both idiopathic and hepatitis C-related thrombocytopenia^21,22^. Unexplained iron deficiency anemia improved with the eradication of *H. pylori* when added to the iron supplement^23^.

Therefore, the present study aimed to estimate frequency and to determine any possible associated risk factors of *H. pylori* infection among a sample of dental students (age 21–25 years) seeking Faculty of Dentistry, Fayoum University. Accordingly, our research question was formulated: what could be the frequency and the possible risk factors associated with Oral *H. pylori* among a sample of dental students?

## Methods

### Following STROBE statement checklist in reporting observational cross-sectional studies

#### Ethics statement

The aim of the study and its benefits were explained to each participant with emphasis on confidentiality of the collected data. Each participant signed an informed consent before being enrolled in the study. The study was approved by the local Ethical Committee at the Faculty of Dentistry, Fayoum University (EC 2217).The study is complying with the Declaration of Helsinki for Medical Research involving Human Subjects.

#### Study design

An observational cross-sectional study.

#### Setting

The study was carried out at the faculty of Dentistry, Fayoum University, Fayoum, Egypt.

#### Participants

Using convenient consecutive sampling technique, 90 dental students were approached to participate. Participants representing the 4th, 5th academic years dental students and also internship, with age ranging from 21 to 25 years old, attending the Faculty of Dentistry, Fayoum University were recruited during a period of 2 months, from November to December 2022.

### Variables and data sources/measurement

#### Outcomes

All the participants were assessed using a structured questionnaire to collect information on the following: age, sex, height, weight, body mass index (calculated as weight/height^2^ (kg/m^2^)), nationality, native region, socioeconomic class, living conditions during childhood (childhood in rural or in urban zone), birth place, number of members of family that sleep in a room and if any of them suffered from *H. pylori* infection, relatives of gastric cancer, non-steroidal anti-inflammatory drugs (NSAIDs) consumption, smoking, spicy food consumption, outdoor food consumption, also GIT involvement symptoms for all participants: upper abdominal pain (epigastric pain, burning), abdominal discomfort (flatulence, bloating), nausea/vomiting and frequent burping, diarrhea, any co-morbid diseases^4^ and also dental history were recorded including oral hygiene status and any oral or dental diseases.

All Participants were subjected to a screening test for anti-*H. pylori* namely enzyme-linked immunosorbent assay (ELISA test) through collecting about 2 to 3 mL of unstimulated whole saliva from them in the saliva collecting vials and assessing them for *H. pylori* immunoglobulins in the clinical laboratory at the Faculty of Medicine. Those with positive results were informed of the necessity to carry out a confirmatory test (PCR) to confirm the diagnosis of *H. pylori* infection and seek medical management.

The primary outcome was the estimation of the frequency of *H. pylori* infection among a sample of dental students. The secondary outcome was the identification of the potential risk factors for *H. pylori* infection among these students at the Faculty of Dentistry, Fayoum University.

### Detection of human *H. pylori* antibody IgG (HP Ab IgG) in saliva by ELISA kit

This kit is provided by Bioassay Technology Laboratory BT LAB (Zhejiang, China) with catalogue number (ED4123Hu). This kit is based on a Qualitative reverse phase enzyme immunoassay technique. The microtiter plate has been pre-coated with a target antigen. Positive/Negative Controls or samples are added to the wells and incubate. Antibodies in the samples bind to the antigen on the plate. Unbound antibody is washed away during a washing step. A Horseradish Peroxidase (HRP) conjugated detection antibody is then added and incubate. Unbound HRP is washed away during a washing step. TMB substrate is then added and color develops. The reaction is stopped by addition of acidic stop solution and color changes into yellow that can be measured at 450 nm. The OD of an unknown sample can then be compared to the OD of the positive and negative controls in order to determine the presence of Hp Ab IgG.

### Sample size

Sample size is calculated based on the previous work by Lin et al.^7^ and Liu et al.^6^ the reported prevalence of *H. pylori* was 16%. Using power 80% and 5% significance level with 90% CI and 180 total students’ number; 81 participants were required. Sample size is calculated using Epi Info.

### Statistical methods

All the results were subjected to statistical analysis. Statistical analysis was done using Statistical Package for Social Sciences, Version 27.0 (SPSS, IBM) for Windows. Continuous variables were summarized as mean values ± standard deviation (SD) or median (range) and compared among groups using Independent t test or Mann Whitney as appropriate. Percentages were calculated for categorical data and comparison was done using Chi square and fisher exact test as appropriate.

### Ethics approval and consent to participate

The aim of the study and its benefits were explained to each participant with emphasis on confidentiality of the collected data. Each participant signed an informed consent before being enrolled in the study. The study was approved by the local Ethical Committee at the Faculty of Dentistry, Fayoum University (EC 2217).The study is complying with the Declaration of Helsinki for Medical Research involving Human Subjects.

## Results

### Characteristics of participants

Eighty-three participants, from which 66 were Egyptians, and 17 were non-Egyptians including 2 Jordanians, 5 Palestinians, 2 Sudanese, 7 Syrians and only 1 Yemeni. Fifty one participants (61.40%) were females. As regards the academic grade, participants from the 4th grade represented 32.50% while the 5th year and internship represented 30.10% and 37.30% respectively. All the participants were resident in Fayoum.

44.58% of our sample were living in rural zone during childhood. The majority of our participants reported medium socioeconomic status [73(88.0%)] with median one person and range from one to nine family members sleep in the same room as shown in (Table 1).

**Table 1:** Characteristics of partcipants.

		n (%)
Age (yrs.)	Mean ± SD	22.9 ± 1.4
	Range	20–29
Gender	Female	51 (61.4)
	Male	32 (38.6)
BMI (kg/m^2^)	Mean ± SD	24.2 ± 4.3
	Range	17.9–43.0
Nationality	Egyptian	66 (79.5)
	Non-Egyptian	17 (20.5)
Special habits	Smoking	13 (15.7)
Academic Grade	Year 4	27 (32.5)
	Year 5	25 (30.1)
	Internship	31 (37.3)
Living conditions during childhood	Rural	37 (44.6)
	Urban	46 (55.4)
Socioeconomic status (Self-reported)	High	10 (12.0)
	Medium	73 (88.0)
Number of members of family that sleep in a room	Median (Range)	1 (1–9)

*SD* standard deviation, *BMI* body mass index.

### *H. pylori* history, symptoms and prevalence

Positive history of *H. pylori* infection was reported by only 12 participants (14.5%). A family history of *H. pylori* infection was reported in two participants while gastric cancer was only recorded in 5 participants, all of whom were representing first degree relatives.

Direct inquiry using assessment questionnaire about dyspeptic symptoms was done on all the 83 enrolled participants and 21 (25.3%) of them presented with more than two of the clinical characteristics., epigastric pain/burning was the most common (25.30%; 21/83), followed by diarrhea (24.10%; 20/83), bloating (22.90%; 19/83), nausea/vomiting (21.69%; 18/83) and frequent burping (8.43%; 7/83). Of 83 participants, 19 were *H. pylori* positive, giving an overall frequency of 22.9% (Table 2).

**Table 2:** History of past infection of *H. pylori* and GIT symptoms.

		n (%)
History of *H. pylori* infection	Positive	12 (14.5)
Family History of *H. pylori* infection	Positive	2 (2.40)
Family History of gastric cancer	Positive	5 (6.0)
History of GIT symptoms	Positive	21 (25.3)
Current GIT symptoms number	None	53 (63.9)
	One or two	9 (10.8)
	More than two	21 (25.3)
GIT symptoms nature	Epigastric pain/burning	21 (25.3)
	Diarrhea	20 (24.1)
	Bloating	19 (22.9)
	Nausea & Vomiting	18 (12.0)
	Frequent Burping	7 (8.4)
H pylori by ELISA	Positive	19 (22.9)

*GIT* gastro intestinal tract.

### Factors associated with *H. pylori*

Demographic characteristics (age, gender, BMI, nationality and socioeconomic status) were comparable between *H. pylori* positive and negative participants. Participants in internship were more prone to be positive (41.9%) in comparison to those whom were in 4th (7.4%) and 5th year (16.0%) with p value = 0.005.

Concerning the living conditions during childhood, 32.6% of urban residents versus 10.8% of rural residents were *H. pylori* positive and this was statistically significant (*p* = 0.019). Regarding previous history of *H. pylori* infection, 75.0% of history of *H. pylori* infection versus 14.1% of those with no history of *H. pylori* infection were positive for *H. pylori* ELIZA test in our study which was found to be statistically significant *p* < 0.001.

For H pylori positive participants about 70% or more reported positive clinical symptoms and were all statistically significant (Table 3).

**Table 3:** Comparison between *H. Pylori* status regarding participants characteristics and history of previous infection.

		*H. pylori* status		*P* value
		Negative	Positive	
		n = 64(%)	n = 19(%)	
Age(yrs.)	Mean ± SD	22.9 ± 1.5	22.7 ± 1.2	0.529
	Range	20–29	21–25	
Gender	Female	39 (76.5)	12 (23.5)	0.861
	Male	25 (78.1)	7 (21.9)	
BMI (kg/m^2^)	Mean ± SD	24.3 ± 4.6	23.5 ± 2.8	0.440
	Range	17.9–43.0	18.9–31.0	
Nationality	Egyptian	50 (75.8)	16 (24.2)	0.564
	Non-Egyptian	14 (82.4)	3 (17.6)	
Socioeconomic status	High	7 (70.0)	3 (30.0)	0.568
	Medium	57 (78.1)	16 (21.9)	
Academic Grade	Year 4	25 (92.6)	2 (7.4)	0.005
	Year 5	21 (84.0)	4 (16.0)	
	Internship	18 (58.1)	13 (41.9)	
Living conditions during childhood	Rural	33 (89.2)	4 (10.8)	0.019
	Urban	31 (67.4)	15 (32.6)	
History of *H. pylori* infection	No	61 (85.9)	10 (14.1)	< 0.001
	Yes	3 (25.0)	9 (75.0)	
Family History of H pylori infection	No	63 (77.8)	18 (22.2)	0.408^a^
	Yes	1 (50.0)	1 (50.0)	
Family History gastric cancer	No	62 (79.5)	16 (20.5)	0.076^a^
	Yes	2 (40.0)	3 (60.0)	
History of GIT signs or symptoms	No	55 (88.7)	7 (11.3)	< 0.001
	Yes	9 (42.9)	12 (57.1)	
Epigastric pain/burning	No	59 (95.2)	3 (4.8)	< 0.001
	Yes	5 (23.8)	16 (76.2)	
Diarrhea	No	58 (92.1)	5 (7.9)	< 0.001
	Yes	6 (30.0)	14 (70.0)	
Bloating	No	58 (90.6)	6 (9.4)	< 0.001
	Yes	6 (31.6)	13 (68.4)	
Nausea & Vomiting	No	59 (90.8)	6 (9.2)	< 0.001
	Yes	5 (27.8)	13 (72.2)	
Frequent Burping	No	62 (81.6)	14 (18.4)	0.001
	Yes	2 (28.6)	5 (71.4)	

Any oral or dental diseases and oral hygiene status were comparable between *H. pylori* positive and negative participants with no statistical significance (Table 4).

**Table 4:** Comparison between *H. Pylori* status regarding Oral hygiene, oral diseases and personal habits.

		*H_pylori*_ELISA		*P* value
		Negative	Positive	
		n = 64(%)	n = 19(%)	
Any Oral or Dental diseases	No	29 (70.7)	12 (29.3)	0.172
	Yes	35 (83.3)	7 (16.7)	
Previous dental treatment	No	31 (68.9)	14 (31.1)	0.052
	Yes	33 (86.8)	5 (13.2)	
Type of treatment	Scaling	4 (80.0)	1 (20.0)	NA
	Restorative Material	18 (94.7)	1 (5.3)	
	Extraction	8 (80.0)	2 (20.0)	
	Root Canal	2 (66.7)	1 (33.3)	
	Ortho	1 (100.0)	0 (.0)	
Oral hygiene status	Excellent	3 (100.0)	0	0.214
	Good	50 (79.4)	13 (20.6)	
	Moderate	11 (64.7)	6 (35.3)	
Smoking	No	55 (78.6)	15 (21.4)	0.462
	Yes	9 (69.2)	4 (30.8)	
Excessive spicy food consumption	No	43 (78.2)	12 (21.8)	0.744
	Yes	21 (75.0)	7 (25.0)	
Excessive outdoor food consumption (junk/fast food/unhealthy)	No	34 (82.9)	7 (17.1)	0.213
	Yes	30 (71.4)	12 (28.6)	
Mouth breathing	No	53 (76.8)	16 (23.2)	0.886
	Yes	11 (78.6)	3 (21.4)	
Daily tooth brushing	No	5 (83.3)	1 (16.7)	1.000^a^
	Yes	59 (76.6)	18 (23.4)	
How many times brushing teeth per day?	None	5 (83.3)	1 (16.7)	0.445
	Once	32 (84.2)	6 (15.8)	
	Twice	24 (68.6)	11 (31.4)	
	3 Times	3 (75.0)	1 (25.0)	
Frequency	None	5 (83.3)	1 (16.7)	NA
	Regular	57 (77.0)	17 (23.0)	
	Irregular	2 (66.7)	1 (33.3)	
The use of other oral hygiene devices	No	52 (81.3)	12 (18.8)	0.099
	Yes	12 (63.2)	7 (36.8)	

## Discussion

*H. pylori* infection is one of the most common chronic bacterial infections worldwide. It is considered a major threat to public health that has been linked to fatal consequences^24^. Confirmed cases of *H. pylori* infection is predicated on isolation, culture, and identification of the bacteria. Because of the difficulty and expense of this process, simpler noninvasive procedures are urgently needed especially in areas with little resources, such as rural and semiurban communities^10^. Salivary ELISA had diagnostic values comparable to serum ELISA, suggesting it might be utilized as a substitute for the latter in the diagnosis of *H. pylori* infection^11^.

Unfortunately, there is scarcity of published data in Egypt in the scientific literature especially concerning dentists, dental students and dental nurses. The population-based prevalence of *H. pylori* infection in Egypt is estimated to be between 26 and 90%^19^.

In the present study, we evaluated the seroprevalence of *H. pylori* infection among dental students aged from 21 to 25 years old, attending the Faculty of Dentistry, Fayoum University,using saliva samples, and we found a frequency of 22.9% (24.2% of Egyptians were *H. pylori* positive versus 17.6% of Non-Egyptians but Arabic population) and 77.1% were *H. pylori* negative, which is regarded to be lower than the previously indicated prevalence range in adults in Egypt, and which may be explained by higher socioeconomic status within our research group, notwithstanding the constraints of our sample size and screening test.

Abdelmonem et al.^25^ also found an overall seroprevalence of *H. pylori* of 52% in Egypt’s Delta area. They reported a higher prevalence of *H. pylori* among adults than children.

In a study to detect *H. pylori*–related iron deficiency anemia prevalence among asymptomatic cases of anaemia in selected private laboratories in the governorates of Beheira, Alexandria and Gharbiya. 180 out of 300 cases of iron deficiency anaemia (60%) tested positive for *H. pylori*^26^. It should be noted that Hamed et al.^27^ also analyzed the frequency of *H. pylori* infection among Egyptian cirrhotic patients in Zagazig University Hospital, and it was found to be 58%. Therefore, the recent decrease in *H. pylori* seroprevalence rate in the present study (22.9%), which may indicate a falling *H. pylori* seroprevalence in Egypt which can be explained by better standards of living related to our selected study population despite the limitations of our sample size and screening test.

In comparison with other studies conducted among dentists, our rate (22.9%) was found to be higher than that reported in a cross-sectional study carried out by Liu, et al.^6^ who found that 7.27% of saliva samples from the nondentist group (N = 110) and 16.67% of saliva samples from the dentist group (N = 90) were oral *H. pylori* positive, and the difference between the two groups was statistically significant (χ2 = 4.292, *p* = 0.038). He reported that even after stratifying enrolled subjects with factors interfering with the comparison of *H. pylori* detection rate between groups, a higher *H. pylori* frequency was observed in the dentists than that in the controls. Also, Lin, et al.^7^ reported that the prevalence of *H. pylori* infection in dentists, dental nurses, fifth year dental students and first year dental students were 23%, 18%, 18%and 16%, respectively, which is considered lower than ours. This indicates that dentists and dental students (21–25 years) in Egypt are at a higher risk for *H. pylori* infection, and more attention should be given to *H. pylori* prevalence studies among them.

Comparing our rate to that of a study carried out among college students in Yuncheng in China, wherein Yu and Zhang^28^ found that the oral *H. pylori* prevalence among these students was 51.25%. In addition, our incidence was lower than that seen in a research conducted in India among 30 dental students aged 20–25, whereby Neeharika^8^ reported 25 positive cases from saliva samples.

We found that our prevalence rate was lower than the rates in several other countries including a study conducted in Thailand’s Khon Kaen Province, where the prevalence of *H. pylori* was found in the saliva of 55% of asymptomatic people between the ages of 18 and 80^29^. Furthermore, in 2018, the total prevalence of *H. pylori* in northeastern Thailand was 64% of the asymptomatic study group (aged 18–60), as detected by testing their saliva^12^ . Positive rates for oral *H. pylori* were 59.59% in Beijing, China, among asymptomatic people aged 20–45^30^. While, 49.6% of the population has *H. pylori* infection in Iran’s Shahid Beheshti hospital in Kashan^11^. In addition, 64.39% were *H. pylori* seropositive people in Cameroon, ranging in age from 35 to 75^31^with the limitations of our sample age range (21–25 years).

The demographic features of our research sample (including age, gender, BMI, nationality, socioeconomic level and frequency of toothbrushing) were comparable between *H. pylori* positive and negative participants, corroborated the findings of a research conducted in northeastern Thailand by Wongphutorn et al.^12^ who observed no correlation between *H. pylori* colonization and demographic variables such as age, gender, employment or frequency of toothbrushing. In addition, as indicated by Piroozmand et al.^11^ it was found no statistically significant association between age and *H. pylori* infection (*p* = 0.8).

Higher rates of positivity were seen among participants in their internship year (41.9%) compared to those in their fourth (7.4%) and fifth (16.0%) years (*p* = 0.005), suggesting that greater vulnerability to seropositive *H. pylori* develops with age. This agrees with what was proposed by Abdelmonem et al.^25^ who found that the seroprevalence of *H. pylori* was greater among adults than children.

Urbanites were more likely to test positive for *H. pylori* (32.6%) than rurality’s (10.8%) due to differences in their early environments, which was a statistically significant result (*p* = 0.019).This finding corroborated the findings of Diab et al.^32^ who found that 85% (51/60) of the positive *H. pylori* patients were from urban regions while 15% (9/60) were from rural areas. But, Awdalla et al.^33^ found that 28% of the urban group was *H. pylori* positive versus 54% in the rural group, which runs counter to our own data. Authors of this Egyptian research found a statistically significant link between *H. pylori* as well as sloppy hygiene practices.

Regarding previous history of *H. pylori* infection, 75% of history of *H. pylori* infection versus 14.1% of those with no history of *H. pylori* infection were positive for *H. pylori* ELIZA test in our study which was found to be statistically significant *p* < 0.001. This result was in line with what had been shown in other research about the frequency with which *H. pylori* recurrences occurred (20–26% and 22% respectively)^4,10,15,16,32^.

In contrast to the work of Kouitcheu Mabeku et al.^31^ who showed that low income and family history of gastric cancer were risk factors for *H. pylori* in his sample population, we discovered no statistically significant association between a family history of gastric cancer and the development of the *H. pylori* infections in our research.

Any oral or dental diseases and oral hygiene status were comparable between *H. pylori* positive and negative participants, which contradicted a research conducted on the Chinese population by Ding et al.^34^ who found that oral *H. pylori* infection is common in adult Chinese, and is significantly associated with oral diseases.

Twenty-one (25.3%) of the 83 participants who filled out the assessment questionnaire, had more than two clinical characteristics associated with dyspepsia. Epigastric pain/burning was the most common (25.30%; 21/83), followed by diarrhea (24.10%; 20/83), bloating (22.90%; 19/83), nausea/vomiting (21.69%; 18/83) and frequent burping (8.43%; 7/83). More than 70% of those who tested positive for *H. pylori*, also reported positive clinical symptoms; these were all statistically significant. As a result, the association between *H. pylori* and the clinical symptoms of the research population has been established. Epigastric discomfort or burning, diarrhea, bloating, nausea, vomiting and frequent burping are all symptoms strongly associated with *H. pylori* infection among the participants, corroborated the findings of Brigitte et al.^4^ who found that all patients experiencing upper stomach discomfort and frequent burping were infected with *H. pylori*.

In conclusion, results from our investigation indicated that the prevalence of *H. pylori* in our sample group was lower than what had been reported for population-based prevalence studies in Egypt but higher than that reported among dentists and dental students in some countries, indicating that dentists and dental students (21–25 years) in Egypt are at a higher risk for *H. pylori* infection, and more care should be given to this matter. People of both low and moderate incomes, previous history of *H. pylori*, clinical symptoms such as epigastric discomfort or burning, diarrhea, bloating, nausea or vomiting and frequent burping were risk factors for *H. pylori* infection amongst these people. Additionally, our research showed that there is no convincing evidence that *H. pylori* infection leads to an increased risk of developing oral illnesses. Therefore, more attention should be given to *H. pylori* prevalence studies among dentists and dental students for better understanding of its epidemiological aspects leading better management of the infection.

## Data Availability

The data used to support the findings of this study are included in this published article.
